# Low-Cost Reliable Corrosion Sensors Using ZnO-PVDF Nanocomposite Textiles

**DOI:** 10.3390/s21124147

**Published:** 2021-06-17

**Authors:** Tonoy Chowdhury, Nandika D’Souza, Narendra Dahotre

**Affiliations:** 1 Department of Mechanical Engineering, University of North Texas, Denton, TX 76207, USA; tonoychowdhury@my.unt.edu; 2 Department of Material Science and Engineering, University of North Texas, Denton, TX 76207, USA; narendra.dahotre@unt.edu

**Keywords:** nanocomposite, electrospinning, ZnO-PVDF fiber, corrosion, sensor, thermal cycling

## Abstract

Submerged steel pipes are susceptible to corrosion due to long exposure under harsh corrosive conditions. Here, we investigated the reliability and effectiveness of nonwoven zinc(II) oxide-polyvinylidene fluoride (ZnO-PVDF) nanocomposite fiber textiles as an embedded corrosion sensor. An accelerated thermal cyclic method paired to electrochemical impedance spectroscopy (EIS) was used for this purpose. Sensor accuracy and reliability were determined using the textile and instrument as reference electrodes. The results showed that the coating and the sensor improved the corrosion resistance when ZnO was added to the sensor textile and introduced into the coating. As the coating’s glass transition was approached, the corrosion performance of the coating degraded and the sensor accuracy decreased. The results suggested that the flexible sensor is reliable at both monitoring the corrosion and acting as a corrosion barrier.

## 1. Introduction

Metals have a tendency to corrode on exposure to the environment especially where there is high moisture content. The situation becomes worse in the case of submerged steel pipes exposed to saltwater. Organic coatings serve as a barrier between the metal substrate and the surrounding environment to reduce the transport of moisture, water, and ionic species to the metal substrate [1]. However, after being exposed to the corrosive environment for a long time, these organic coatings start degrading and cause metal corrosion. Corrosion sensors have been investigated using an embedded electrode or sensor placed in the coating to measure changes in the electrochemical properties [2,3,4,5]. Researchers have embedded platinum, gold, and nickel sensor layers at the metal–coating interface to measure the real-time electrochemical impedance [5,6,7,8,9,10,11]. A major drawback in such a system is the corrosion of the sensing element itself as it is also metallic. Moreover, cost becomes a concern for wide-area application and measurement of the corrosion response. To overcome these limitations, a low-cost nonconductive sensing material is highly desired in order to detect corrosion without being corroded. In our previous work, we have shown that a nonconductive ZnO-PVDF nanocomposite nonwoven textile, fabricated from the electrospinning method, could be used as an embedded sensor to sense corrosion under the organic coating after it was immersed into a sea salt solution [12]. The textile was found to offer two benefits. First, it performed well as a sensor and the impedance matched that of an external calibrated corrosion instrument. Second, the presence of the textile improved the corrosion performance of the coating by increasing the corrosion resistance. In this paper, we investigated the long-term reliability of the sensor through accelerated aging and the impact of the coating–glass transition on the sensor’s performance.

The long-term reliability of corrosion sensors can be best determined by the examination of the sensor under actual service conditions [13]. However, the time needed for the degradation of insulative coatings with slow diffusion rates renders that option impractical. To counter that, accelerated methods such as Prohesion or salt fog have been introduced, which can simulate coating service conditions and promote failure in shorter times [14]. To promote that, the samples are mounted in a chamber where a saltwater solution is atomized by spray nozzles using pressurized air for both tests. Prohesion differs from the salt fog tests in that the salt solution used in the Prohesion test is much more dilute (5% NaCl for salt fog and 0.05% NaCl and 0.35% (NH₄)₂SO₄ for Prohesion), and exposure is intermittent [15,16]. For both tests, it takes months to make small changes in most polymer coating systems. Even with higher-concentration salt, researchers have shown that both the low-frequency impedance modulus and noise resistance remained almost unchanged after 2 years of exposure of the polyurethane/epoxy primer coating system over 2024 aluminum alloy for the salt fog experiment [17,18]. In response to the need to develop an accurate and accelerated test, a thermal cycling approach emerged. In this method, the coated panels are subjected to cyclic temperature changes under constant immersion. The method involves heating the coating to an elevated temperature related to the coating–glass transition followed by cooling to room temperature concomitant to the monitoring of impedance changes through each transition. Researchers have determined that thermal cycling induces irreversible changes on the coatings. The deviation in low-frequency impedance as a function of cycles between ambient and elevated temperatures is used as an indicator of coating failure [19,20]. The failure in the coatings has been shown to replicate the degradation mechanisms as the changes caused by natural atmospheric conditions occurring over longer times [19,21,22,23,24].

In this paper, a nonconductive sensing textile, ZnO-PVDF mesh, was used as an embedded sensor to overcome the limits of the highly conductive sensing elements that amplify the corrosion of the pipe. Textiles as a sensing element will also be facile to incorporate into the corrosion pipe coating manufacturing process as pipe cladding with fabric paired to the coating application on pipes is a large-scale manufacturing solution currently used. One could also anticipate that the introduction of a textile will create a physical barrier that will make the coating resistant to surface scratches that can create fast path zones for permeating fluids. To test the reliability of the sensor, an accelerated thermal cycling method was used under constant immersion in 4.2% sea salt solution. The diffusion of the electrolyte solution into the epoxy coating can be expected to be minimal at temperatures well below *T_g_*. As the temperature of the epoxy coating approaches *T_g_*, the increase in fluid permeation into the epoxy coating through diffusion can no longer be ignored. The set points were thus chosen to the coating *T_g_*. As the sensor textile polymer, PVDF, has a *T_g_* of around 160 °C, which is much higher than that of the epoxy coating (55 °C), the sensor will have limited independent material contributions to the exposure of temperatures indexed to the *T_g_* of the epoxy coating. We selected ZnO nanoparticles as they are hydrophobic and thermally stable nanoparticles for the composite sensor textile [25,26]. EIS testing was carried out for the textile as a stand-alone system, as well as embedded at the metal–coating interface to evaluate the performance of a standard epoxy coating, along with the sensor’s capability of sensing corrosion when exposed to the thermal cycling method. Three cycles for each elevated set point temperature were chosen, enabling the determination of the cyclic reliability of the sensor as a function of cycle number. The temperature dependence of the barrier properties of the coating, along with its activation energy as a function of ZnO, were calculated by examining the reversibility of impedance data.

## 2. Materials and Methods

**Figure 1:**
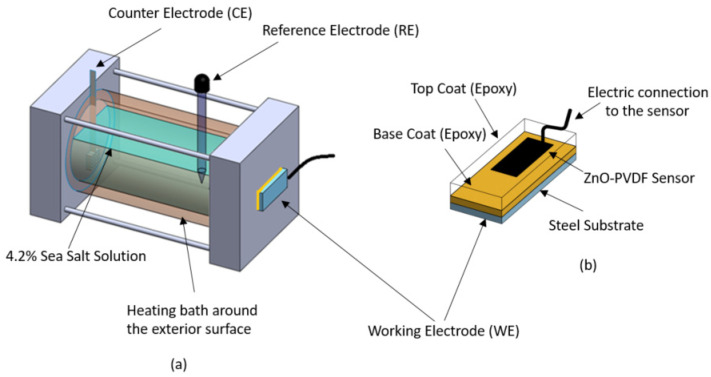
Schematic diagram of (**a**) double jacket corrosion cell for EIS testing under thermal cyclic conditions and (**b**) double-layer epoxy-coated steel substrate with embedded sensor. EIS experimental setup: Configuration A (actual data): steel substrate is WE, Pt mesh is CE, and standard calomel electrode is RE; Configuration B (sensor data): steel substrate is WE, Pt mesh is CE, and sensor is RE.

The ZnO-PVDF fiber mats were fabricated using the electrospinning process. To prepare the corrosion sensor sample, the steel coupons were first coated with an epoxy coating with a thickness of 120 ± 10 µm. Then, the ZnO-PVDF fiber mat was placed on the coating with copper core electrical wire connected to it, followed by a second layer of the same epoxy coating (120 ± 10 µm), as shown in Figure 1. The electrospinning process and corrosion sample preparation process have been described in our previous work [12]. To establish the validity of the sensor, EIS testing was carried out on two different configurations to confirm the accuracy of the textile as a sensor. This is described in our previous work [12]: Configuration A (instrument data) and Configuration B (sensor data). In Configuration A, the coated steel substrate operated as a working electrode (WE), the Pt mesh as the counter electrode (CE), and the standard calomel electrode as the reference electrode (RE), whereas the steel substrate worked as the WE, the Pt mesh as the CE, and the sensor textile as the RE in Configuration B.

### Thermal Cycling Method

**Figure 2:**
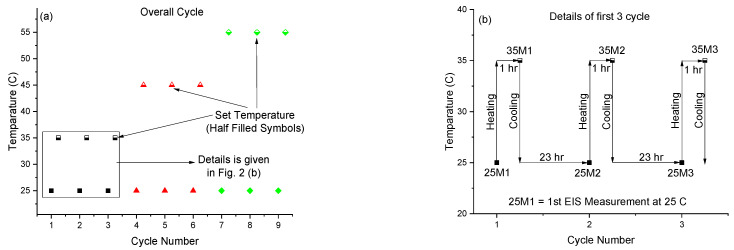
Schematic of thermal cycling method: (**a**) temperature as a function of cycle number used in the experiment and (**b**) detailed experimental procedure for first 3 cycles.

The thermal cycling experiment was conducted using a flat corrosion testing cell with a 1 cm^2^ sample area. The cell was obtained from Biologic and has a double jacket for temperature control. First, the coated panel was immersed in 4.2% sea salt solution at room temperature for 1 day until a steady state was reached, after which the thermal cycling measurements were initiated. The thermal cycle method consisted of nine cycles with three different set temperatures, 35, 45, and 55 °C, which corresponded to values of (*T_g_* −20 °C), (*T_g_* −10 °C), and *T_g_*, as shown in Figure 2a. Three cycles were run at each set temperature of 35, 45, and 55 °C, and each cycle consisted of two EIS measurements: one at room temperature and the other at the set temperature. For example: in cycle 1, the first EIS measurement of the sample was taken at room temperature (25M1), and then the sample was heated to the set temperature (35 °C) for 1 h using a heating bath (35M1) around the exterior surface of the double jacket corrosion cell, followed by a second cycle of 25M2 and 35M2 and third cycle of 25M3 and 35M3. This is shown in Figure 2b. Table 1 shows the indexing used in this experiment. We use *X*M*Y*, where M stands for the EIS measurement, *X* is the temperature of the EIS measurement, and *Y* is the cycle number. All notations for the 9 measurements for each sensor textile are tabulated in Table 1.

## 3. Results and Discussion

### 3.1. EIS Results

**Figure 3:**
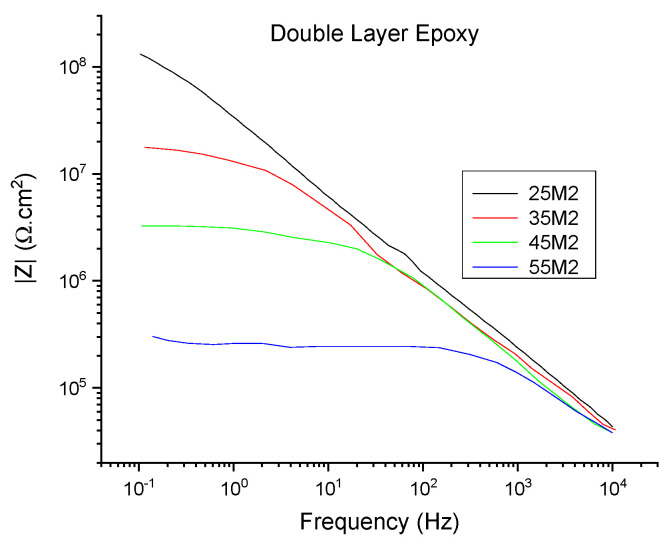
Bode impedance data for double-layer epoxy at thermal cyclic method.

**Figure 4:**
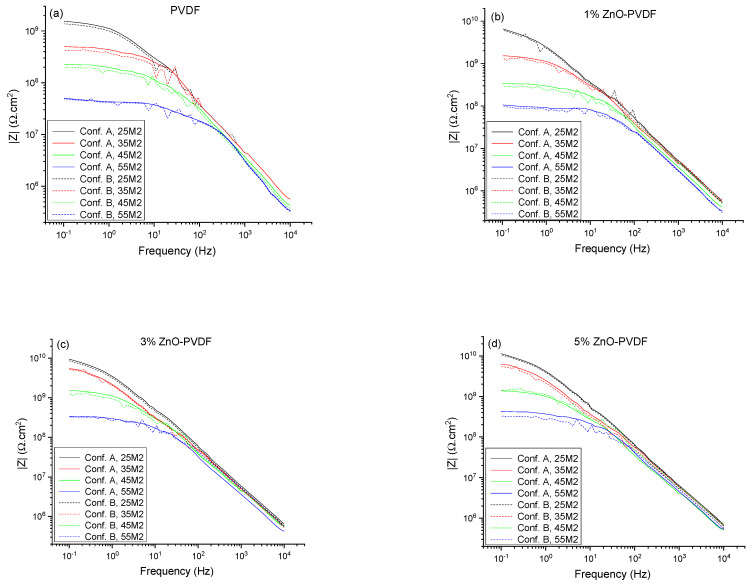
Bode magnitude plots at different temperatures for (**a**) PVDF fiber, (**b**) 1% ZnO-PVDF fiber, (**c**) 3% ZnO-PVDF fiber, and (**d**) 5% ZnO-PVDF fiber. The solid line is associated with the instrument data and the dotted line represents the sensor data. The 25 °C data are associated with the 2nd EIS measurement at cycle 2 (25M2), while the 35, 45, and 55 °C data are associated with the 2nd EIS measurement of the three cycles to which the coating was exposed (35M2, 45M2, and 55M2, respectively).

In order to determine the efficiency of the sensor in real-time monitoring, corrosion was induced through the thermal cycling method, which involved exposing the coating to alternating room temperature and higher temperature (35, 45, and 55 °C or (*T_g_* −10 °C), (*T_g_* −10 °C), and *T_g_*) cycles to perform EIS testing using Configurations A and B. The Bode plot of the topcoat/basecoat system with the epoxy coating alone is shown in Figure 3, and those for the different sensor textiles are shown in Figure 4 associated with Configuration A and B, where the 25 °C data are related to cycle 2 (25M2) and the set temperature data are related to the second of the three cycles that the coating was subjected to (35M2, 45M2, and 55M2). At 25 °C (*T_g_* −30 °C), the impedance values *|Z|* of PVDF, 1% ZnO-PVDF, 3% ZnO-PVDF, and 5% ZnO-PVDF fiber embedded samples were 1.53 GΩ, 6.55 GΩ, 9.24 GΩ, and 11.3 GΩ, respectively, indicating a sharp increase in corrosion resistance after adding ZnO into the fiber. This was due to the addition of hydrophobic ZnO to PVDF fiber, which increased the resistance and capacitance of the coating [25,27]. For reference, the epoxy alone has an impedance modulus of 0.078 GΩ, indicating textiles contribute substantially to improving the corrosion resistance in coatings.

**Figure 5:**
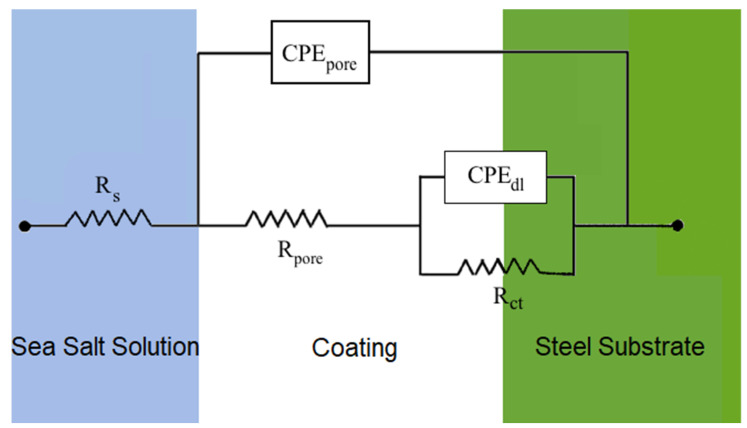
Electrochemical equivalent circuit from EIS data [12].

On the other hand, the EIS data of set temperatures showed a plateau region while transitioning from high to low frequency. This became more apparent at 45 °C (*T_g_* −10 °C) and higher temperatures. This indicated that the corrosion resistance (e.g., pore and charge transfer resistance) of the coating started degrading significantly after exposing it to 45 °C (*T_g_* −10 °C). Typically, the epoxy impedance remained unchanged at room temperatures with low humidity. As the temperature increased in the presence of an electrolyte solution, the barrier properties, along with other mechanical properties such as flexural and compressive strength of the epoxy, decreased. The reason for this effect is that the epoxy adsorbs the electrolyte and ions from the surrounding environment. This transport of electrolyte through the epoxy coating becomes more apparent when the temperature increases. When the temperature approaches the *T_g_* of epoxy, it absorbs water into it rapidly [19,20]. In our case, the epoxy started and accumulated more water in it at 45 °C (*T_g_* −10 °C), thereby reducing the corrosion resistance significantly. The plateau region became more dominant for 55 °C (*T_g_*) data, which is consistent with the previous discussion. The overlay of the Bode plots of Configuration A and B also showed similar trends, indicating the efficiency of the sensor. For additional analysis, the EIS data, shown in Figure 4 and Figure S1, were fitted into an electrochemical equivalent circuit (EEC) using EC-Lab software, as shown in Figure 5, which was previously validated for insulative coatings [28,29]. In this proposed EEC, *R_s_* is the solution resistance, and *R_pore_*, *CPE_pore_*, *R_ct_*, and *CPE_dl_* are the pore resistance, pore capacitance, charge transfer resistance, and double-layer capacitance of the system, respectively. A constant phase element (*CPE*) was used in the EEC instead of pure capacitance *C* to counter the surface heterogeneity of the coating, and its impedance is defined as *Z_CPE_* = *Z_0_/(jω)^n^*, where *Z_0_* and *n* are constant [30]. The average of the fitted value of all the EIS measurements for each parameter is listed in Table 2. The 25 °C data are the average of each circuit parameter extracted from EIS measurements of the 1st three cycles (25M1, 25M2, and 25M3), and the 35, 45, and 55 °C data are associated with the average of the three cycles to which the coating was exposed. The results in Table 2 were found to be consistent with the Bode magnitude plot, as shown in Figure 4. The chi-square (*χ2*) from the fitting results was on the order of 10^−2^, indicating the goodness of the curve fitting.

Comparing *R_pore_* values between Configuration A and B of the PVDF fiber from Table 2, it is clear that the percentage of error was less than 10% up to 35 °Cv (*T_g_* −20 °C). As the temperature increased, *R_pore_* decreased and percentage of error increased. This indicates that the coating was tough until 35 °C (*T_g_*−20 °C). As the temperature increased, electrolytes entered into the coating and reached the sensor textile, causing the increase in error percentage. Other EEC parameters such as *C_pore_*, *R_ct_*, and *C_dl_* followed the same trend. However, the 1%, 3%, and 5% ZnO-PVDF sensor textiles showed an error less than 10% until 45 °C (*T_g_* −10 °C). This happened due to the introduction of the hydrophobic ZnO into the sensor textile, which resisted the electrolyte entering into the coating up to 45 °C (*T_g_* −10 °C). As the temperature approached the *T_g_* (55 °C) of the coating, the electrolyte penetrated through the coating and reached the sensor textile, and the percentage of error jumped to approximately 20% for all the EEC parameters. It is also notable that the error between the sensor data and instrumental data reduced after the addition of ZnO in PVDF fiber mats, indicating an improvement in the reliability of the sensor.

### 3.2. Effect of Cyclic Temperature Exposure

**Figure 6:**
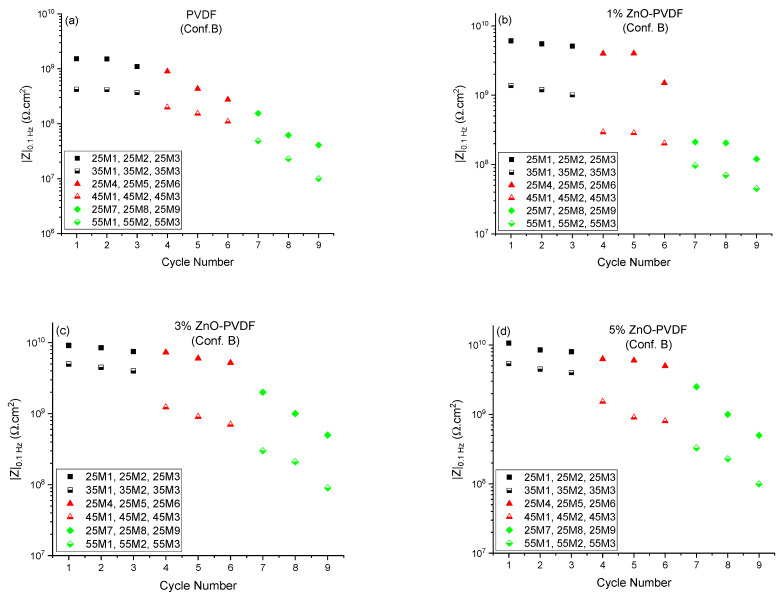
*|Z|*_0.1 *Hz*_ values measured at room temperature and set temperatures as a function of thermal cycle number for (**a**) PVDF fiber, (**b**) 1% ZnO-PVDF fiber, (**c**) 3% ZnO-PVDF fiber, and (**d**) 5% ZnO-PVDF fiber. The 25M1 to 25M9 data are associated with *|Z|*_0.1 *Hz*_ values measured at 25 °C of each cycle, while the 35M*Y*, 45M*Y,* and 55M*Y* data are associated with set temperatures (35, 45, and 55 °C) where *Y* = 1, 2, and 3.

To better understand the failure process in the coating, a comprehensive study was conducted on the reversibility of the coating’s barrier property. We employed the recommendations of Bierwagen et al. [17] that the barrier property of the coating is equivalent to the d.c. resistance, measured from the low-frequency impedance modulus of EIS data. The Bode magnitude plot shown in Figure 4 has a low-frequency plateau, which is indicative of the d.c. limit of the coating. The impedance at the lowest frequency was selected (*|Z|*_0.1 *Hz*_) from the Bode plot to represent the barrier property of the epoxy coating systems for Configuration B. The *|Z|*_0.1 *Hz*_ values measured at 25 °C and at the set temperatures of the coating system are shown in Figure 6 as a function of cycle number. The 25M1 to 25M9 data are associated with *|Z|*_0.1 *Hz*_ values measured at 25 °C of each cycle, while the 35M*Y*, 45M*Y*, and 55M*Y* data are associated with the set temperatures (35, 45, and 55 °C) where *Y* = 1, 2, and 3.

For the PVDF fiber embedded coating, as shown in Figure 6a, the *|Z|*_0.1 *Hz*_ values at 25 °C were roughly the same at 1.5 X 10^9^ Ω.cm^2^ for up to four cycles. Thus, the changes in impedance were induced by the increase in temperature, though it was not permanent for up to four cycles. This showed the reversibility of the coating after being exposed to 35 °C (*T_g_* −20 °C) for three times and 45 °C (*T_g_* −10 °C) for a single time. This happened due to not having any permanent damage on the epoxy coating by thermal cycles. The mobility of polymer segments and the polymer free volume increased with temperature but returned to their original states as the system cooled down to room temperature [20]. Thus, the electrolytes that entered into the epoxy coating were driven out when the temperature returned to 25 °C. The *|Z|*_0.1 *Hz*_ values for the PVDF fiber embedded coating did not return to its initial value from cycle 5; rather, it continued decreasing as the thermal cycle number progressed. This indicated that the reversibility of the coating was limited to a temperature as high as 35 °C (*T_g_* −20 °C). Thermal cycling above that temperature (45 °C and 55 °C or *T_g_* −10 °C and *T_g_*) induced permanent changes to the PVDF fiber embedded epoxy coating under constant immersion in 4.2% sea salt solution. When the ZnO was introduced in the PVDF fiber and embedded between two layers of coating, the permanent change in the epoxy coating was also delayed to cycle 6 for 1% ZnO-PVDF, and cycle 7 for both 3% ZnO-PVDF and 5% ZnO-PVDF instead of cycle 5 for the PVDF fiber, as shown in Figure 6b–d, respectively. This happened due to the addition of thermally stable ZnO particles, which improved the thermal properties of the coating and dropped the degradation rate [26,27,31]. *|Z|*_0.1 *Hz*_ values from Configuration B followed the same trend as those of Configuration A.

### 3.3. Temperature Dependence of EIS Impedance

**Figure 7:**
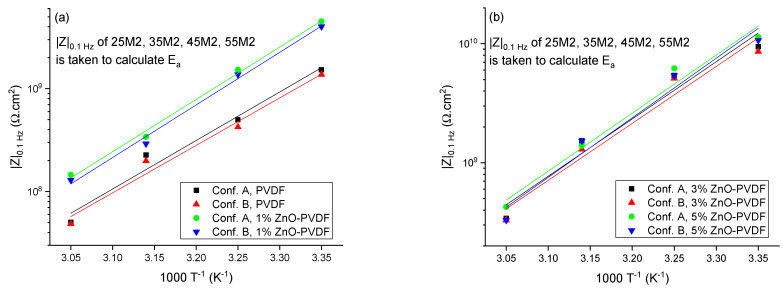
The low-frequency impedance modulus (*|Z|*_0.1 *Hz*_) as a function of 1/*T* for (**a**) PVDF and 1% ZnO-PVDF, and (**b**) 3% ZnO-PVDF and 5% ZnO-PVDF.

The temperature dependence of the impedance of the coatings embedded with different fiber sensors was evaluated by comparing their activation energy. The activation energies were calculated using Equation (1) [8,32] from both Configuration A and B data under the assumption of Arrhenius behavior of the coatings.

(1) $${\left|Z\right|}_{0.1\;Hz}=A\cdot exp\frac{E_{a}}{RT}$$

where *|Z|*_0.1_ *_Hz_* is the impedance of the coating at 0.1 Hz, *E_a_* is the activation energy, *A* is the Arrhenius constant, *T* is the temperature in Kelvin, and *R* is the universal gas constant. The EIS impedance of the coatings *|Z|* at 0.1 Hz, measured from Configuration A and B (25M2, 35M2, 45M2, and 55M2), is shown in Figure 7 as a function of 1/*T*. There were observable linear relationships between the logarithmic values and 1/*T* for both configurations’ data, indicating that the barrier properties were consistent with the Arrhenius behavior.

Activation energy, *E_a_*, is the minimum amount of energy that is required for atoms or molecules of a system to activate a chemical reaction or physical transport. The larger the *E_a_* value, the better the barrier property of the coating [33,34]. Table 3 lists the magnitude of *E_a_* of the epoxy coatings from both configurations as a function of ZnO mass fraction in the embedded fibers. *E_a_* of the epoxy coating embedded with the PVDF mesh was 55.91 kJ.mol^−1^, and it increased to 61.53 kJ.mol^−1^ as ZnO increased to a 5% mass ratio in the fiber, indicating that the coating became more stable against any chemical reaction or physical transport of corrosive fluid. Both the *E_a_* values obtained from Configuration A and B were consistent with less than 1% error in all cases. It should be noted that the activation energy is not significantly affected by the magnitude of ZnO in the textile, showing that the epoxy (coating) permeability governs the sensor textile performance.

## 4. Conclusions

This paper investigated the reliability of the electrospun ZnO-PVDF mats as an embedded corrosion sensor using accelerated corrosion testing. To accelerate the corrosion in the coating system, the thermal cycling method was used. The results show that the introduction of textiles into epoxy substantially improved the corrosion resistance of the epoxy coating. Further, when ZnO was added, the impedance increased. As a sensor, the accuracy of the corrosion resistance increased over the base PVDF. From the vantage point of a sensor textile, the 3% ZnO-PVDF sensor was most optimum, showing a deviation of less than 10% in resistances for temperatures of 45 °C (*T_g_*−10 °C) and below. As the set temperature of the thermal cycle approached the *T_g_* of the epoxy coating, the barrier property of the coating was damaged significantly and sensor reading deviated. The repeated cycling reliability of the impedance measured in the coating via the instrument and sensor textile was examined. The results indicate that the cyclic retention of the impedance measured increased when ZnO was present in the textile over the unmodified textile. The corrosion performance of the coating and reliability of the sensor textile was reversible up to 35 °C (*T_g_*−20 °C) and 45 °C (*T_g_* −10 °C) for the PVDF fiber and ZnO-PVDF fiber embedded coating, respectively, after which the coating started to degrade through ingestion of the saltwater, and irreversible changes occurred. This improvement in reversibility from the addition of ZnO could be attributed to using thermally stable and hydrophobic ZnO nanoparticles into the PVDF matrix, which improved the thermal and barrier properties of the coating significantly by increasing the activation energy of the system. As a result, the addition of ZnO decreased the measurement error in the sensor textile and improved the coating’s barrier property.

## Figures and Tables

**Table 1:** Notation for the three EIS measurements conducted in thermal cyclic method.

Index	Meaning
25M1, 25M2, 25M3	EIS measurements at room temperature when set temperature is 35 °C (*T_g_* −20 °C)
25M4, 25M5, 25M6	EIS measurements at room temperature when set temperature is 45 °C (*T_g_* −10 °C)
25M7, 25M8, 25M9	EIS measurements at room temperature when set temperature is 55 °C (*T_g_*)
35M1, 35M2, 35M3	EIS measurements at set temperature of 35 °C (*T_g_* −20 °C)
45M1, 45M2, 45M3	EIS measurements at set temperature of 45 °C (*T_g_* −10 °C)
55M1, 55M2, 55M3	EIS measurements at set temperature of 55 °C (*T_g_*)

**Table 2:** Electrochemical equivalent circuit parameters at different temperatures and configurations.

Sample	Conf.	*R_pore_* (MΩ)	*R_ct_* (GΩ)	*C_pore_* (pF.s^n−1^.cm^−2^)	*C_dl_* (µF.s^n−1^.cm^−2^)	Error for *R_pore_* (%)	Error for *R_ct_* (%)	Error for *C_pore_* (%)	Error for *C_ct_* (%)
Pure_Epoxy_25°C	-	0.23	0.12	5.77	0.35	-	-	-	-
Pure_Epoxy_35°C	-	0.19	0.08	5.93	1.66	-	-	-	-
Pure_Epoxy_45°C	-	0.16	0.02	6.75	2.12	-	-	-	-
Pure_Epoxy_55°C	-	0.12	0.001	7.23	2.43	-	-	-	-
PVDF_25°C	A	2.47 ± 0.17	1.40 ± 0.11	5.92 ± 0.12	2.74 ± 0.16	5.34	6.23	1.08	5.12
	B	2.34 ± 0.13	1.31 ± 0.14	5.92 ± 0.20	2.60 ± 0.22				
PVDF_35°C	A	1.09 ± 0.29	0.41 ± 0.12	6.62 ± 0.27	2.98 ± 0.30	9.33	10.75	8.56	6.37
	B	1.01 ± 0.37	0.37 ± 0.25	6.28 ± 0.38	2.79 ± 0.43				
PVDF_45°C	A	0.28 ± 0.79	0.11 ± 0.87	6.91 ± 0.51	3.15 ± 0.55	17.71	19.09	9.11	9.40
	B	0.23 ± 0.68	0.09 ± 0.76	6.28 ± 0.68	2.90 ± 0.82				
PVDF_55°C	A	0.02 ± 1.05	0.03 ± 1.06	7.11 ± 1.82	3.32 ± 1.49	27.12	22.22	10.13	12.65
	B	0.01 ± 1.11	0.01 ± 1.01	6.39 ± 1.79	2.90 ± 1.95				
1%_ZnO_25°C	A	5.21 ± 0.25	5.67 ± 0.23	5.21 ± 0.05	5.45 ± 0.08	4.35	3.27	1.88	2.83
	B	4.98 ± 0.39	5.48 ± 0.21	5.49 ± 0.12	5.29 ± 0.15				
1%_ZnO_35°C	A	2.42 ± 0.16	1.35 ± 0.28	6.01 ± 0.16	5.82 ± 0.02	7.85	6.67	4.16	2.58
	B	2.23 ± 0.27	1.26 ± 0.14	5.76 ± 0.08	5.67 ± 0.57				
1%_ZnO_45°C	A	1.23 ± 0.59	0.25 ± 0.55	6.38 ± 0.81	5.97 ± 0.72	7.57	9.70	8.62	4.02
	B	1.10 ± 0.45	0.22 ± 0.68	6.93 ± 0.49	5.73 ± 0.54				
1%_ZnO_55°C	A	0.05 ± 0.75	0.09 ± 0.75	6.49 ± 0.74	6.25 ± 0.96	21.82	11.11	14.18	20.80
	B	0.04 ± 0.79	0.08 ± 0.89	7.41 ± 0.49	4.95 ± 0.79				
3%_ZnO_25°C	A	7.05 ± 0.29	8.02 ± 0.15	9.68 ± 0.08	5.08 ± 0.22	1.75	3.15	2.80	5.03
	B	6.93 ± 0.17	7.76 ± 0.23	9.41 ± 0.10	4.82 ± 0.09				
3%_ZnO_35°C	A	5.43 ± 0.28	5.23 ± 0.21	9.89 ± 0.17	5.45 ± 0.26	4.42	3.25	4.55	8.44
	B	5.19 ± 0.37	5.06 ± 0.35	9.44 ± 0.15	4.26 ± 0.29				
3%_ZnO_45°C	A	1.93 ± 0.19	1.37 ± 0.27	10.81 ± 0.34	6.69 ± 0.52	8.95	8.14	9.16	10.51
	B	2.18 ± 0.23	1.19 ± 0.31	9.82 ± 0.49	5.92 ± 0.38				
3%_ZnO_55°C	A	0.59 ± 0.74	0.27 ± 0.84	10.98 ± 0.91	7.28 ± 0.71	15.90	19.28	10.66	10.16
	B	0.43 ± 0.87	0.21 ± 0.72	9.81 ± 0.69	6.54 ± 0.65				
5%_ZnO_25°C	A	8.34 ± 0.16	10.27 ± 0.19	10.97 ± 0.24	6.33 ± 0.03	3.93	4.82	3.20	5.96
	B	8.01 ± 0.10	9.78 ± 0.26	10.62 ± 0.20	6.08 ± 0.14				
5%_ZnO_35°C	A	5.86 ± 0.25	6.09 ± 0.23	11.16 ± 0.16	6.65 ± 0.31	4.78	2.79	1.34	5.71
	B	5.58 ± 0.22	5.92 ± 0.17	11.01 ± 0.28	6.27 ± 0.20				
5%_ZnO_45°C	A	2.28 ± 0.36	2.01 ± 0.41	12.62 ± 0.54	7.24 ± 0.47	9.84	9.95	8.56	6.35
	B	2.01 ± 0.47	1.79 ± 0.51	11.54 ± 0.62	6.78 ± 0.23				
5%_ZnO_55°C	A	1.21 ± 0.92	0.59 ± 0.98	12.89 ± 0.79	7.54 ± 0.81	15.70	18.64	9.46	13.93
	B	1.02 ± 0.89	0.48 ± 0.91	11.67 ± 0.65	6.49 ± 0.77				

**Table 3:** Activation energy of different samples at different configurations.

Sample	*E_a_* from Conf. A (kJ.mol^−1^)	*E_a_* from Conf. B (kJ.mol^−1^)	Error (%)
Coating with PVDF	55.91 ± 0.31	55.62 ± 0.31	0.52
Coating with 1% ZnO-PVDF	58.37 ± 0.15	58.05 ± 0.19	0.55
Coating with 3% ZnO-PVDF	60.65 ± 0.38	60.41 ± 0.37	0.41
Coating with 5% ZnO-PVDF	61.53 ± 0.41	61.31 ± 0.48	0.35

## Supplementary Materials

The following are available online at https://www.mdpi.com/article/10.3390/s21124147/s1, Figure S1: Bode phase plots at different temperatures for (**a**) PVDF fiber, (**b**) 1% ZnO-PVDF fiber, (**c**) 3% ZnO-PVDF fiber, and (**d**) 5% ZnO-PVDF fiber. The solid line is associated with the instrument data and the dotted line represents the sensor data. The 25 °C data are associated with the 2nd EIS measurement at cycle 2 (25M2), while the 35, 45, and 55 °C data are associated with the 2nd EIS measurement of the three cycles to which the coating was exposed (35M2, 45M2, and 55M2, respectively).

## References

[1] Augustyniak A., Tsavalas J., Ming W. (2009). Early Detection of Steel Corrosion via “Turn-On” Fluorescence in Smart Epoxy Coatings. ACS Appl. Mater. Interfaces.

[2] Bierwagen G.P., Allahar K.N., Su Q., Gelling V.J. (2009). Electrochemically characterizing the ac-dc-ac accelerated test method using embedded electrodes. Corros. Sci..

[3] Bierwagen G., Wang X., Tallman D. (2003). In situ study of coatings using embedded electrodes for ENM measurements. Prog. Org. Coat..

[4] Kittel J., Celati N., Keddam M., Takenouti H. (2001). New methods for the study of organic coatings by EIS: New insights into attached and free films. Prog. Org. Coat..

[5] Kittel J., Celati N., Keddam M., Takenouti H. (2003). Influence of the coating-substrate interactions on the corrosion protection: Characterisation by impedance spectroscopy of the inner and outer parts of a coating. Prog. Org. Coat..

[6] Allahar K.N., Hinderliter B.R., Bierwagen G.P., Tallman D.E. (2009). Army Vehicle Primer Properties During Wet-Dry Cycling. Corrosion.

[7] Allahar K.N., Su Q., Bierwagen G.P., Lee D.H. (2008). Monitoring of the AC-DC-AC Degradation of Organic Coatings Using Embedded Electrodes. Corrosion.

[8] Su Q., Allahar K.N., Bierwagen G.P. (2008). Application of embedded sensors in the thermal cycling of organic coatings. Corros. Sci..

[9] Su Q., Allahar K., Bierwagen G. (2008). Embedded electrode electrochemical noise monitoring of the corrosion beneath organic coatings induced by ac-dc-ac conditions. Electrochim. Acta.

[10] Allahar K.N., Upadhyay V., Bierwagen G.P., Gelling V.J. (2009). Monitoring of a military vehicle coating under Prohesion exposure by embedded sensors. Prog. Org. Coat..

[11] Miszczyk A., Schauer T. (2005). Electrochemical approach to evaluate the interlayer adhesion of organic coatings. Prog. Org. Coat..

[12] Chowdhury T., D’Souza N., Ho Y.H., Dahotre N., Mahbub I. (2020). Embedded Corrosion Sensing with ZnO-PVDF Sensor Textiles. Sensors.

[13] Wright R.F., Lu P., Devkota J., Lu F., Ziomek-Moroz M., Ohodnicki P.R. (2019). Corrosion Sensors for Structural Health Monitoring of Oil and Natural Gas Infrastructure: A Review. Sensors.

[14] Baer A. In The Effective Use of Epoxy Insulative Coatings in the Oil & Gas Industry. Proceedings of the NACE International Corrosion Conference.

[15] Merino S.F., Caprari J.J., Torres L.V., Ramos L.F., Girola A.H. (2017). Inhibitive action of tara tannin in rust converter formulation. Anti-Corros. Methods Mater..

[16] Bower K., Murray S., Reinhart A., Nieto A. (2020). Corrosion resistance of selective laser melted Ti–6Al–4V alloy in salt fog environment. Results Mater..

[17] Bierwagen G., Tallman D., Li J., He L., Jeffcoate C. (2003). EIS studies of coated metals in accelerated exposure. Prog. Org. Coat..

[18] Allahar K.N., Su Q., Bierwagen G.P. (2008). In Situ Monitoring of Organic Coatings Under QUV/Prohesion Exposure by Embedded Sensors. Corrosion.

[19] Touzain S., Le Thu Q., Bonnet G. (2005). Evaluation of thick organic coatings degradation in seawater using cathodic protection and thermally accelerated tests. Prog. Org. Coat..

[20] Bierwagen G.P., He L., Li J., Ellingson L., Tallman D. (2000). Studies of a new accelerated evaluation method for coating corrosion resistance—Thermal cycling testing. Prog. Org. Coat..

[21] Miszczyk A., Darowicki K. (2001). Accelerated ageing of organic coating systems by thermal treatment. Corros. Sci..

[22] Miszczyk A., Darowicki K. (2003). Effect of environmental temperature variations on protective properties of organic coatings. Prog. Org. Coat..

[23] Valentinelli L., Vogelsang J., Ochs H., Fedrizzi L. (2002). Evaluation of barrier coatings by cycling testing. Prog. Org. Coat..

[24] Richards B.T., Begley M.R., Wadley H.N. (2015). Mechanisms of Ytterbium Monosilicate/Mullite/Silicon Coating Failure During Thermal Cycling in Water Vapor. J. Am. Ceram. Soc..

[25] Zhang X., Si Y., Mo J., Guo Z. (2017). Robust micro-nanoscale flowerlike ZnO/epoxy resin superhydrophobic coating with rapid healing ability. Chem. Eng. J..

[26] Samad U.A., Alam M.A., Chafidz A., Al-Zahrani S.M., Alharthi N.H. (2018). Enhancing mechanical properties of epoxy/polyaniline coating with addition of ZnO nanoparticles: Nanoindentation characterization. Prog. Org. Coat..

[27] Hu C., Li Y., Kong Y., Ding Y. (2016). Preparation of poly(o-toluidine)/nano ZnO/epoxy composite coating and evaluation of its corrosion resistance properties. Synth. Met..

[28] Amirudin A., Thieny D. (1995). Application of electrochemical impedance spectroscopy to study the degradation of polymer-coated metals. Prog. Org. Coat..

[29] Alsamuraee A.M.A., Jaafer H.I. (2011). Electrochemical impedance spectroscopic evaluation of corrosion protection properties of polyurethane /polyvinyl chloride blend coatings on steel. Am. J. Sci. Ind. Res..

[30] Zhang J., Zhang L., Wilke B.M., Li W., Ning C., Chowdhury T. (2017). Corrosion Behavior of Microarc Oxidized Mg Alloy in Earle’s Balance Salt Solution. Surf. Innov..

[31] Ramezanzadeh B., Attar M.M. (2011). Studying the corrosion resistance and hydrolytic degradation of an epoxy coating containing ZnO nanoparticles. Mater. Chem. Phys..

[32] Aneja K.S., Böhm H.M., Khanna A., Böhm S. (2017). Functionalised graphene as a barrier against corrosion. FlatChem.

[33] Li J., Jeffcoate C.S., Bierwagen G.P., Mills D.J., Tallman D.E. (1998). Thermal Transition Effects and Electrochemical Properties in Organic Coatings: Part 1—Initial Studies on Corrosion Protective Organic Coatings. Corrosion.

[34] Sykes J.M., Whyte E.P., Yu X., Sahir Z.S. (2017). Does “coating resistance” control corrosion?. Prog. Org. Coat..

