# Supplementary material for: Low-Cost Reliable Corrosion Sensors Using ZnO-PVDF Nanocomposite Textiles
Source: Sensors (Basel). 2021 Jun 17;21(12):4147. doi: 10.3390/s21124147 (PMC8235672; doi:10.3390/s21124147)
Supplement: Supplementary file 1 [file sensors-21-04147-s001.zip › sensors-1183527-supplementary.pdf]

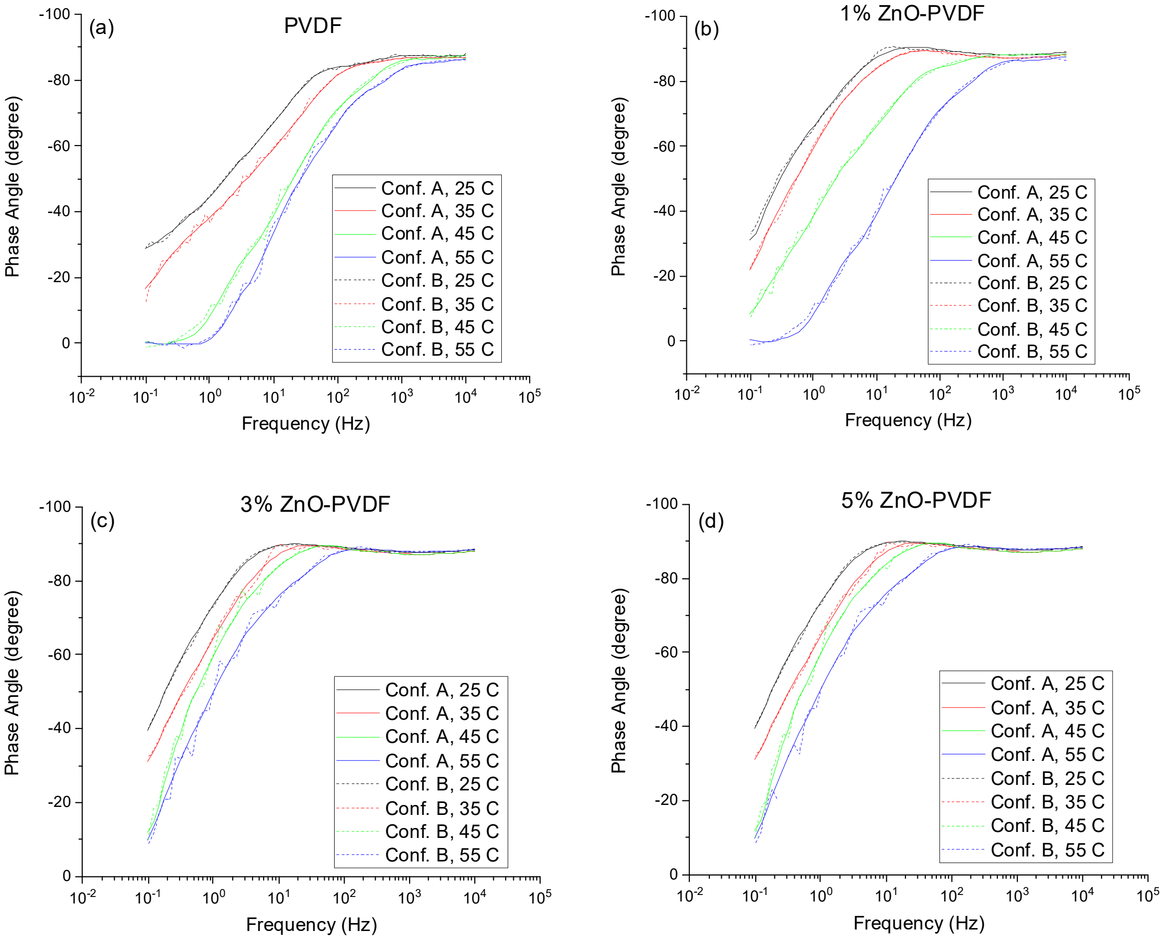

Figure S1. Bode phase plots at different temperature for (**a**) PVDF fiber, (**b**) 1% ZnO-PVDF fiber, (**c**) 3% ZnO-PVDF fiber and (**d**) 5% ZnO-PVDF fiber. Solid line is associated with the instrument data and dotted line represents the sensor data. The 25 ˚C data is associated with 2nd EIS measurement at cycle 2 (25M2) while 35, 45, and 55 ˚C data is associated to the 2nd EIS measurement of the three cycles that the coating was exposed (35M2, 45M2 and 55M2, respectively).
